# *o*-Phenyl­enediaminium chloride nitrate

**DOI:** 10.1107/S1600536813007447

**Published:** 2013-03-28

**Authors:** Sarra Soudani, Riadh Kefi, Christian Jelsch, Emmanuel Wenger, Cherif Ben Nasr

**Affiliations:** a Laboratoire de Chimie des Matériaux, Faculté des Sciences de Bizerte, 7021 Zarzouna, Tunisia; b Cristallographie, Résonance Magnétique et Modélisations (CRM2), UMR CNRS–UHP 7036, Institut Jean Barriol, Université de Lorraine, BP 70239, Boulevard des Aiguillettes, 54506 Vandoeuvre-les-Nancy, France

## Abstract

In the title mol­ecular salt, C_6_H_10_N_2_^2+^·NO_3_^−^·Cl^−^, the complete cation is generated by a crystallographic mirror plane. The complete nitrate ion is also generated by reflection, with the N atom and one O atom lying on the mirror plane; the chloride ion also lies on the reflection plane. In the crystal, the components are linked by N—H⋯Cl and N—H⋯(N,O) hydrogen bonds, forming (001) layers with the benzene rings projecting into the inter­layer regions. The layers are linked by weak C—H⋯O hydrogen bonds, generating a three-dimensional network.

## Related literature

For background to inorganic–organic hybrid compounds, see: Bringley & Rajeswaram (2006 ▶); Dai *et al.* (2002 ▶). For reference structural data, see: Riahi *et al.* (2012 ▶); Engh & Huber (1991 ▶).
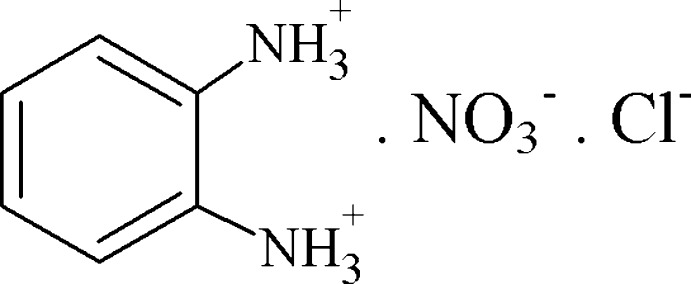


## Experimental

### 

#### Crystal data

C_6_H_10_N_2_^2+^·Cl^−^·NO_3_^−^
*M* *_r_* = 207.61
Orthorhombic, 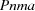

*a* = 7.3695 (5) Å
*b* = 8.2367 (5) Å
*c* = 14.2398 (7) Å
*V* = 864.36 (8) Å^3^
*Z* = 4
Ag *K*α radiation
λ = 0.56085 Å
μ = 0.22 mm^−1^
*T* = 100 K
0.27 × 0.20 × 0.15 mm

#### Data collection

Bruker Photon100 CMOS detector diffractometer
Absorption correction: multi-scan (*SADABS*; Bruker, 2004 ▶) *T*_min_ = 0.751, *T*_max_ = 0.967
26823 measured reflections
812 independent reflections
812 reflections with *I* > 2σ(*I*)
*R*_int_ = 0.049

#### Refinement

*R*[*F* ^2^ > 2σ(*F* ^2^)] = 0.024
*wR*(*F* ^2^) = 0.053
*S* = 0.80
812 reflections
66 parameters
12 restraints
H atoms treated by a mixture of independent and constrained refinement
Δρ_max_ = 0.27 e Å^−3^
Δρ_min_ = −0.26 e Å^−3^

### 

Data collection: *COLLECT* (Bruker, 2004 ▶); cell refinement: *SCALEPACK* (Otwinowski & Minor, 1997 ▶); data reduction: *DENZO* (Otwinowski & Minor, 1997 ▶) and *SCALEPACK*; program(s) used to solve structure: *SIR92* (Altomare *et al.*, 1994 ▶); program(s) used to refine structure: *MoPro* (Jelsch *et al.*, 2005 ▶); molecular graphics: *DIAMOND* (Brandenburg, 1998 ▶); software used to prepare material for publication: *publCIF* (Westrip, 2010 ▶).

## Supplementary Material

Crystal structure: contains datablock(s) global, I. DOI: 10.1107/S1600536813007447/hb7052sup1.cif

Structure factors: contains datablock(s) I. DOI: 10.1107/S1600536813007447/hb7052Isup2.hkl

Supplementary material file. DOI: 10.1107/S1600536813007447/hb7052Isup3.cml

Additional supplementary materials:  crystallographic information; 3D view; checkCIF report

## Figures and Tables

**Table 1:** Hydrogen-bond geometry (Å, °)

*D*—H⋯*A*	*D*—H	H⋯*A*	*D*⋯*A*	*D*—H⋯*A*
N3—H34⋯O10	1.033 (4)	2.41 (1)	2.896 (2)	107.4 (9)
N3—H34⋯N1	1.033 (4)	2.429 (9)	3.263 (2)	137.1 (8)
N3—H31⋯Cl1^i^	1.033 (4)	2.181 (4)	3.179 (2)	161.8 (5)
N3—H32⋯Cl1	1.033 (4)	2.183 (5)	3.156 (2)	156.2 (5)
C2—H2⋯O11	1.08	2.48	3.299 (2)	132
C1—H1⋯O10^ii^	1.08	2.52	3.427 (2)	141

Symmetry codes: (i) 
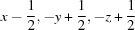
; (ii) 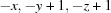.

**Fig. 1.**
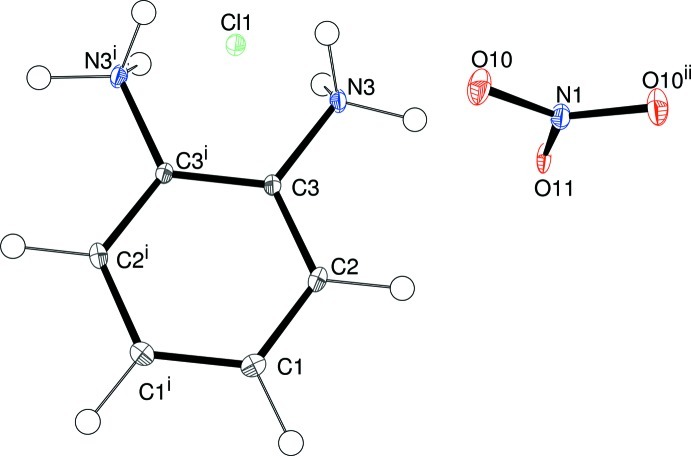
A view of the title compound, showing 50% probability displacement ellipsoids and spheres for the H atoms. Symmetry codes: (i) x, 1/2-y, z; (ii) x, 3/2-y, z.

**Fig. 2.**
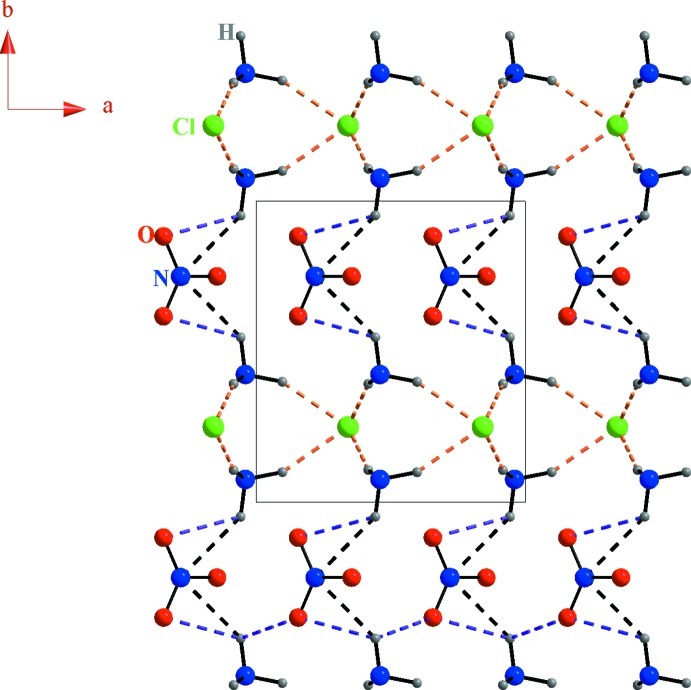
Projection along the c-axis of the inorganic layers in the structure of the title compound. Hydrogen bonds are shown as broken lines.

**Fig. 3.**
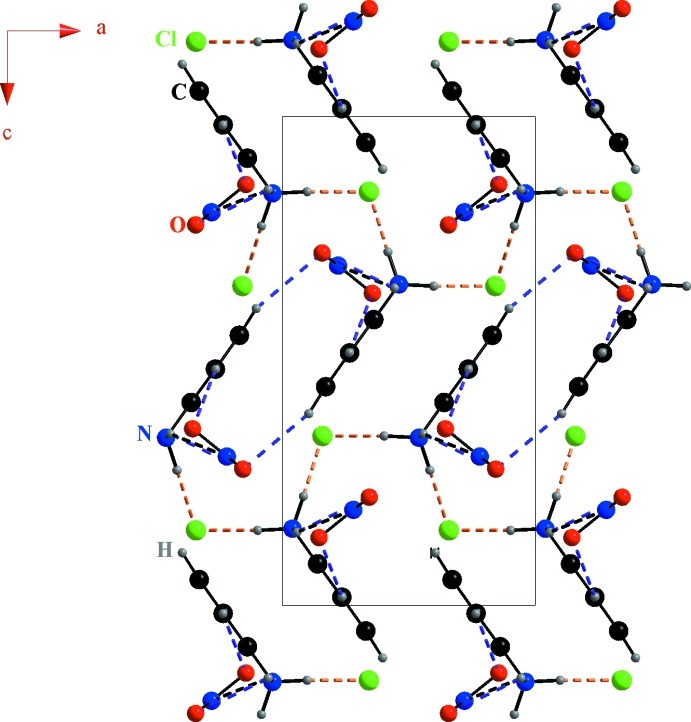
The packing diagram of the compound viewed down the b-axis. Hydrogen bonds are shown as broken lines.

## supplementary crystallographic information

### Comment

Inorganic-organic hybrid compounds provide a class of materials with interesting potential technological applications (Bringley & Rajeswaram 2006; Dai *et al.*, 2002). As part of our studies in this area, we report here the synthesis and the crystal structure of the title compound, (I), formed by the reaction between benzene-1,2-diamine, nitric acid and hydrochloric acid. The structure consists of one nitrate anion, one chloride anion and one benzene-1,2-diaminium dication (Fig. 1). In the crystal, the H atoms of the ammonium groups are involved in two kinds of hydrogen bonds: N—H···Cl and N3—H34···(N1, O10), which is a bifurcated H-bond interaction (Table 1). These hydrogen bonds link the ionic units (NH_3_^+^, Cl^-^ and NO_3_^-^) into layers parallel to (001) (Fig. 2) and situated at *z* = *n* +/- 1/4 (Fig. 3). The chloride ion is actually located on a special position with *y* = 1/4. The phenyl groups of the benzene-1,2-diaminium dications are located alternatively on either side of the ionic layers *via* three weak C—H···O hydrogen bonds (Fig. 3) with the nitrate anion, which is perpendicular to the phenyl plane. No π-π stacking interactions between the organic rings or C—H···π interactions towards them are observed.

The geometrical parameters of the title compound are in the normal range. A N—O moiety of the nitrate is located on a special position *y*=3/4 and the two independant N—O bond distances are 1.243 (2) and 1.284 (1) Å. In addition, the O—N—O bond angle values are 118.6 (2)° and 122.8 (2), showing that the nitrate anion exhibits a slightly distorted C_3 h_ geometry. These anionic geometrical features are comparable to those previously reported for 2-cyanoanilinium nitrate where the N—O bond length distances are in the range 1.228 (2)–1.273 (2) Å and the values of the O—N—O angles are between 117.52 (2) and 121,80 (15)° (Riahi *et al.*, 2012). For the organic cation, the mean value of the C—C bond lengths of the aromatic ring is 1.391 (2) Å which is close to the 1.382 (3) value between >CH aromatic atoms in the Engh & Huber (1991) stereochemical dictionary.

### Experimental

A mixture of an aqueous solution of benzene-1,2-diamine (3 mmol), nitric acid (3 mmol) and hydrochloric acid (3 mmol) was slowly evaporated at room temperature over several days leading to formation of transparent light brown prismatic crystals (yield 60%). The crystals are stable for months under normal conditions of temperature and humidity.

### Crystal data

**Table:**

C_6_H_10_N_2_^2+^·Cl^−^·NO_3_^−^	*F*(000) = 432
*M**_r_* = 207.61	*D*_x_ = 1.596 Mg m^−^^3^
Orthorhombic, *P**n**m**a*	Ag *K*α radiation, λ = 0.56085 Å
Hall symbol: -P 2ac 2n	Cell parameters from 64 reflections
*a* = 7.3695 (5) Å	θ = 3.1–20.3°
*b* = 8.2367 (5) Å	µ = 0.22 mm^−^^1^
*c* = 14.2398 (7) Å	*T* = 100 K
*V* = 864.36 (8) Å^3^	Prismatic, yellow
*Z* = 4	0.27 × 0.20 × 0.15 mm

### Data collection

**Table:**

Bruker Photon100 CMOS detector diffractometer	812 reflections with *I* > 2σ(*I*)
Radiation source: fine-focus sealed tube	*R*_int_ = 0.049
ω scans	θ_max_ = 19.5°, θ_min_ = 2.5°
Absorption correction: multi-scan (*SADABS*; Bruker, 2004)	*h* = 0→8
*T*_min_ = 0.751, *T*_max_ = 0.967	*k* = 0→9
26823 measured reflections	*l* = 0→16
812 independent reflections	

### Refinement

**Table:**

Refinement on *F*^2^	Primary atom site location: structure-invariant direct methods
Least-squares matrix: full	Secondary atom site location: difference Fourier map
*R*[*F*^2^ > 2σ(*F*^2^)] = 0.024	Hydrogen site location: inferred from neighbouring sites
*wR*(*F*^2^) = 0.053	H atoms treated by a mixture of independent and constrained refinement
*S* = 0.80	*w* = 1/[σ^2^(*F*_o_^2^) + (0.*P*)^2^ + 2.3*P*] where *P* = (*F*_o_^2^ + 2*F*_c_^2^)/3
812 reflections	(Δ/σ)_max_ = −0.002
66 parameters	Δρ_max_ = 0.27 e Å^−^^3^
12 restraints	Δρ_min_ = −0.26 e Å^−^^3^

### Special details

**Table:**

Refinement. Refinement of F^2^ against reflections. The threshold expression of F^2^ > 2sigma(F^2^) is used for calculating R-factors(gt) and is not relevant to the choice of reflections for refinement. R-factors based on F^2^ are statistically about twice as large as those based on F, and R-factors based on ALL data will be even larger.

### Fractional atomic coordinates and isotropic or equivalent isotropic displacement parameters (Å^2^)

**Table:**

	*x*	*y*	*z*	*U*_iso_*/*U*_eq_	
Cl1	0.84182 (9)	0.25000	0.34646 (4)	0.00937 (9)	
N1	0.2199 (3)	0.75000	0.3046 (2)	0.0091 (3)	
O11	0.3554 (3)	0.75000	0.3614 (1)	0.0098 (3)	
O10	0.1566 (2)	0.6174 (2)	0.27870 (9)	0.0143 (2)	
C1	0.1707 (3)	0.3346 (2)	0.5526 (1)	0.0117 (3)	
H1	0.10400	0.40337	0.60752	0.01410*	
C2	0.2654 (3)	0.4193 (2)	0.4834 (1)	0.0100 (2)	
H2	0.26439	0.55074	0.48291	0.01199*	
C3	0.3611 (2)	0.3345 (2)	0.4152 (1)	0.0071 (2)	
N3	0.4604 (2)	0.4241 (2)	0.3431 (1)	0.0083 (2)	
H31	0.417 (1)	0.393 (1)	0.2766 (3)	0.01240*	
H32	0.5979 (4)	0.400 (1)	0.3466 (10)	0.01240*	
H34	0.443 (2)	0.5479 (3)	0.3506 (9)	0.01240*	

### Atomic displacement parameters (Å^2^)

**Table:**

	*U*^11^	*U*^22^	*U*^33^	*U*^12^	*U*^13^	*U*^23^
Cl1	0.0105 (3)	0.0079 (3)	0.0097 (3)	0	−0.0003 (3)	0
N1	0.011 (1)	0.006 (1)	0.010 (1)	0	−0.0011 (9)	0
O11	0.0117 (9)	0.0032 (9)	0.0145 (10)	0	−0.0032 (8)	0
O10	0.0168 (7)	0.0076 (6)	0.0184 (7)	−0.0016 (6)	−0.0052 (6)	−0.0023 (6)
C1	0.0149 (9)	0.0101 (10)	0.0102 (9)	0.0008 (8)	0.0030 (8)	−0.0012 (8)
C2	0.0128 (9)	0.0058 (9)	0.0113 (9)	0.0008 (8)	0.0011 (7)	−0.0014 (7)
C3	0.0084 (9)	0.0052 (9)	0.0079 (9)	−0.0001 (8)	0.0010 (7)	0.0003 (7)
N3	0.0099 (8)	0.0049 (7)	0.0100 (7)	−0.0008 (6)	0.0002 (6)	0.0008 (6)

### Geometric parameters (Å, º)

**Table:**

N1—O10	1.243 (2)	C2—H2	1.083
N1—O11	1.284 (3)	C3—N3	1.461 (2)
C1—C2	1.395 (3)	N3—H32	1.033 (4)
C1—H1	1.083	N3—H34	1.033 (4)
C2—C3	1.389 (3)	N3—H31	1.033 (4)
			
O11—N1—O10	118.6 (2)	C3—N3—H32	111.2 (7)
O10—N1—O10^i^	122.8 (2)	C3—N3—H34	111.3 (7)
C1—C2—C3	119.8 (2)	C3—N3—H31	111.1 (6)
C1—C2—H2	120.1	H31—N3—H32	107.7 (8)
H1—C1—C2	118.4	H31—N3—H34	107.6 (9)
C2—C3—N3	119.5 (2)	H32—N3—H34	107.7 (10)
H2—C2—C3	120.1		
			
C1—C2—C3—N3	−179.8 (2)	C2—C3—N3—H34	−2.1 (7)
H1—C1—C2—C3	176.3	C2—C3—N3—H31	−122.0 (7)
H1—C1—C2—H2	−3.7	H2—C2—C3—N3	0.2
C2—C3—N3—H32	118.0 (8)		

Symmetry code: (i) *x*, −*y*+3/2, *z*.

### Hydrogen-bond geometry (Å, º)

**Table:**

*D*—H···*A*	*D*—H	H···*A*	*D*···*A*	*D*—H···*A*
N3—H34···O10	1.033 (4)	2.41 (1)	2.896 (2)	107.4 (9)
N3—H34···N1	1.033 (4)	2.429 (9)	3.263 (2)	137.1 (8)
N3—H31···Cl1^ii^	1.033 (4)	2.181 (4)	3.179 (2)	161.8 (5)
N3—H32···Cl1	1.033 (4)	2.183 (5)	3.156 (2)	156.2 (5)
C2—H2···O11	1.08	2.48	3.299 (2)	132
C1—H1···O10^iii^	1.08	2.52	3.427 (2)	141

Symmetry codes: (ii) *x*−1/2, −*y*+1/2, −*z*+1/2; (iii) −*x*, −*y*+1, −*z*+1.
